# Using the 3D Facial Norms Database to investigate craniofacial sexual dimorphism in healthy children, adolescents, and adults

**DOI:** 10.1186/s13293-016-0076-8

**Published:** 2016-04-22

**Authors:** Matthew J. Kesterke, Zachary D. Raffensperger, Carrie L. Heike, Michael L. Cunningham, Jacqueline T. Hecht, Chung How Kau, Nichole L. Nidey, Lina M. Moreno, George L. Wehby, Mary L. Marazita, Seth M. Weinberg

**Affiliations:** Department of Anthropology, University of Pittsburgh, Pittsburgh, PA USA; Center for Craniofacial and Dental Genetics, Department of Oral Biology, University of Pittsburgh, 100 Technology Drive, Suite 500, Pittsburgh, PA 15219 USA; Department of Pediatrics, University of Washington, Seattle, WA USA; Department of Biological Structure, University of Washington, Seattle, WA USA; Department of Oral Biology, University of Washington, Seattle, WA USA; Department of Pediatric Dentistry, University of Washington, Seattle, WA USA; Department of Pediatrics, University of Texas Health Science Center, Houston, TX USA; Department of Orthodontics, University of Alabama, Birmingham, AL USA; Department of Pediatrics, University of Iowa, Iowa City, IA USA; Department of Orthodontics, University of Iowa, Iowa City, IA USA; Dows Institute for Dental Research, University of Iowa, Iowa City, IA USA; Department of Health Management and Policy, University of Iowa, Iowa City, IA USA; Department of Human Genetics, University of Pittsburgh, Pittsburgh, PA USA; Clinical and Translational Science Institute, University of Pittsburgh, Pittsburgh, PA USA; Department of Psychiatry, University of Pittsburgh, Pittsburgh, PA USA

**Keywords:** Sexual dimorphism, Anthropometry, Stereophotogrammetry, Geometric morphometrics, Facial shape

## Abstract

**Background:**

Although craniofacial sex differences have been extensively studied in humans, relatively little is known about when various dimorphic features manifest during postnatal life. Using cross-sectional data derived from the 3D Facial Norms data repository, we tested for sexual dimorphism of craniofacial soft-tissue morphology at different ages.

**Methods:**

One thousand five hundred fifty-five individuals, pre-screened for craniofacial conditions, between 3 and 25 years of age were placed in to one of six age-defined categories: early childhood, late childhood, puberty, adolescence, young adult, and adult. At each age group, sex differences were tested by ANCOVA for 29 traditional soft-tissue anthropometric measurements collected from 3D facial scans. Additionally, sex differences in shape were tested using a geometric morphometric analysis of 24 3D facial landmarks.

**Results:**

Significant (*p* < 0.05) sex differences were observed in every age group for measurements covering multiple aspects of the craniofacial complex. The magnitude of the dimorphism generally increased with age, with large spikes in the nasal, cranial, and facial measurements observed after puberty. Significant facial shape differences (*p* < 0.05) were also seen at each age, with some dimorphic features already present in young children (eye fissure inclination) and others emerging only after puberty (mandibular position).

**Conclusions:**

Several craniofacial soft-tissue sex differences were already present in the youngest age group studied, indicating that these differences emerged prior to 3 years of age. The results paint a complex and heterogeneous picture, with different groups of traits exhibiting distinct patterns of dimorphism during ontogeny. The definitive adult male and female facial shape was present following puberty, but arose from numerous distinct changes taking place at earlier stages.

**Electronic supplementary material:**

The online version of this article (doi:10.1186/s13293-016-0076-8) contains supplementary material, which is available to authorized users.

## Background

Characterizing sexual dimorphism of the human craniofacial complex is of interest to numerous fields, including physical anthropology [1–3], forensics [4, 5], cognitive science [6, 7], plastic and reconstructive surgery [8], and orthodontics [9–12]. While reduced compared to many other primate species, biological sex accounts for a sizeable portion of craniofacial variation in humans. A recent study in a large sample of racially admixed adults by Claes et al. [13] estimated that biological sex accounted for 12.9 % of the among-individual variation present in facial surface shape, while ancestry accounted for only 9.6 % of shape variation. Such craniofacial sex differences are multifactorial in nature and result from a combination of inherent genetic factors, hormonal influences (operating during both prenatal and postnatal life), and functional demands [14, 15]. While most studies in the literature focus on sex differences involving the skull, sexual dimorphism in craniofacial soft tissue has also been extensively demonstrated in adults [7, 13, 16–23]. From these studies, the general consensus is that adult male faces are larger and characterized locally by more prominent nasal, supraorbital, and chin regions and less prominent orbital, malar, and forehead regions.

Fewer studies have examined craniofacial sexual dimorphism in subadults. One reason for this may be the widely held view that prior to the onset of puberty, there are few, if any, meaningful sex differences in human facial features [14, 24]. Nevertheless, craniometric and cephalometric studies have reported evidence of sex differences in skull morphology during early postnatal life [10, 25, 26]. In a longitudinal growth study, Bulygina et al. [26], for example, showed differences in craniofacial shape using lateral cephalograms as early as the first year of life. These skeletal findings suggest that sex differences in the overlying soft-tissue morphology are likely also present prior to puberty.

A number of studies have explicitly assessed soft tissue craniofacial sex differences in subadults [5, 6, 8–10, 27–41]. Farkas [28] compared a variety of anthropometric proportions in children between 6 and 18 years; notably, the most prominent sex differences at both age six and age 18 involved measures of the lips and mouth. In a longitudinal study, Gaži-Čoklica et al. [11] reported sex differences in several anthropometric measures in children as young as 4.7 years of age; these included measures of the cranial vault width and length and facial height. Studies by Ferrario et al. [8, 29] have demonstrated differences in soft-tissue facial dimensions between boys and girls as young as 6 years of age, most conspicuously in the lower third of the face. Toma et al. [34] compared the average 3D facial surface between males and females in a large sample of 15-year-old children from the UK; the major facial differences included more prominent central facial structures (including the nose and mouth) and less pronounced eyes and cheeks in males. Their results largely replicated the findings from Hennessy et al. [7] and Kau et al. [21] in adults, suggesting that the adult pattern of facial dimorphism is already present around the time of puberty. Sforza and colleagues [36, 37] described sex-specific growth patterns for several nasal and labial dimensions, indicating that the timing of dimorphism varies by trait. More recently, in a longitudinal 3D facial dataset of 12–15 year olds from the Czech Republic, Koudelová et al. [38] reported sex differences in facial shape starting at age 14, although these were mostly size related. Males were characterized by the typical adult pattern of reduced eyes and cheeks and more prominent noses, chins, and brow ridges.

For craniofacial clinicians, understanding of how the soft tissues of the head and face differ between healthy males and females is important because these structures are the ultimate targets, directly or indirectly, of orthodontic and surgical interventions. Because the growth trajectories vary across different portions of the craniofacial complex [42], delineating at what point during the lifespan, various sexually dimorphic facial features emerge is a key concern. Unfortunately, most studies assessing craniofacial sex differences in soft-tissue features are not adequately equipped to address this issue. In studies that have included subadults, the range of available ages has typically been limited, the number of measurements included have been few, the samples included have been small, and/or the methods used failed to adequately take into account overall body size differences. To identify when various sexually dimorphic facial characteristics arise, detailed morphological data covering a wide range of subadult ages are required, including data on very young children. In a recent paper [43], we introduced the 3D Facial Norms (3DFN) repository. Among other data types, this web-based repository contains a variety of craniofacial measurements derived from 3D facial surface images of over 2400 healthy participants between the age of 3 and 40 years (more details can be found at www.facebase.org/facial_norms). In the current study, we use the 3DFN repository to test for sex differences in craniofacial morphology in a large subset of individuals, ranging from early childhood through young adulthood. To provide a comprehensive picture of the nature of facial sexual dimorphism at different life stages, we employ both a traditional linear distance approach and multivariate statistical shape analysis. We predict that different features of the craniofacial complex will demonstrate specific (non-uniform) patterns of sexual dimorphism, with some traits emerging very early in life and others (e.g., mandibular prominence) emerging only after puberty.

## Methods

The study sample was comprised of 1555 individuals (646 males and 909 females) between the ages of 3 and 25 years of age. The age range was selected to capture the youngest participants available in the dataset through the period of young adulthood, when craniofacial growth has largely ceased [14]. All individuals were recruited as part of the 3DFN project, which was limited to individuals of self-identified European ancestry who had no history of craniofacial trauma, congenital malformation, or surgery. Participants were recruited at four US sites: Pittsburgh, Seattle, Houston, and Iowa City. The predominant methods of recruitment were targeted advertisement and institutional research registries. Appropriate institutional ethics (IRB) approval was obtained at each recruitment site. A detailed account of the recruitment methods and eligibility criteria for the project are provided in our previous report [43]. Prior to analysis, individuals were separated by age into six distinct groups [44]: early childhood (3–6 years of age), late childhood (7–12 years of age), puberty (13–15 years of age), adolescence (16–18 years of age), young adult (19–21 years of age), and adult (22–25 years of age). Descriptive statistics for each age group are provided in Table 1.

**Table 1:** Basic descriptive statistics on the study sample

	Males					Females				
Age group (years)	*N*	Mean age	SD	Mean height	SD	*N*	Mean age	SD	Mean height	SD
Early childhood (3–6)	98	4.39	1.12	110.1	9.8	95	4.38	1.18	109.2	10.7
Late childhood (7–12)	128	9.49	1.82	141.8	14.0	118	9.76	1.67	141.7	13.0
Puberty (13–15)	52	13.89	0.83	168.9***	9.2	56	13.79	0.85	161.8	7.0
Adolescence (16–18)	46	17.43	0.83	180.3***	9.2	65	17.63	0.67	166.5	7.5
Young adult (19–21)	98	20.12	0.84	180.9***	8.4	215	20.01	0.82	167.0	6.4
Adult (22–25)	222	23.55	1.08	182.2***	7.1	360	23.46	1.09	165.7	6.9
Total	646					909				

Significant difference between males and females indicated as *p ≤ 0.05, **p ≤ 0.01, ***p ≤ 0.001. Age reported in years. Height reported in cm

**Fig. 1:**
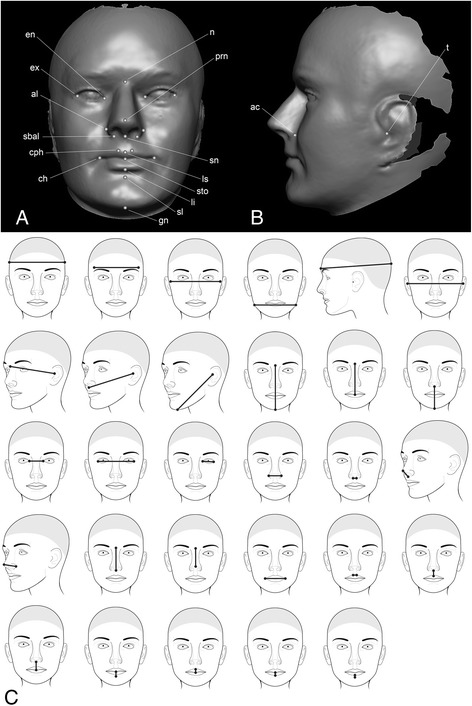
Facial landmarks and anthropometric measurements used in the study. Parts (**a**) and (**b**) show an example of a 3D facial surface model with the 24 standard landmarks used in the shape analysis. Landmarks shown in frontal view (**a**) are *n* nasion; *prn* pronasale; *sn* subnasale; *ls* labiale superius; *sto* stomion; *li* labiale inferius; *sl* sublabiale; *gn* gnathion; *en* endocanthion; *ex* exocanthion; *al* alare; *sbal* subalare; *cph* crista philtri; *ch* chelion (for bilateral points, only right side is labeled). Landmarks shown in the lateral view (**b**) are *ac* alar curvature point and *t* tragion (only left landmark shown for these two bilateral points). Part (**c**) shows the approximate location of the 29 linear distances listed in Table 2. From top left to bottom right, these are as follows: maximum cranial width, minimum frontal width, maximum facial width, mandibular width, maximum cranial length, cranial base width, upper facial depth (*left*), middle facial depth (*left*), lower facial depth (*left*), morphological facial height, upper facial height, lower facial height, intercanthal width, outercanthal width, palpebral fissure length (*left*), nasal width, subnasal width, nasal protrusion, nasal ala length (*left*), nasal height, nasal bridge length, labial fissure width, philtrum width, philtrum length, upper lip height, lower lip height, upper vermilion height, lower vermilion height, and cutaneous lower lip height. The first five measurements were collected manually with spreading calipers. The remaining 24 measurements were calculated from the 3D landmarks shown in parts (**a**) and (**b**)

For the traditional morphometric analysis, 29 anthropometric linear distances covering the entire craniofacial complex were included (Table 2 and Fig. 1). Five of these distances were collected using direct anthropometry with spreading calipers (GPM, Switzerland). The remaining 24 distances were derived from 3D facial surfaces obtained using digital stereophotogrammetry (3dMD; Atlanta, GA). These 24 distances were calculated from landmarks collected on 3D facial surfaces and correspond to traditional anthropometric measurements [45, 46] and are shown in Fig. 1. The measurement error associated with facial landmark collection for the 3DFN project has been previously described [43], with intraclass correlation coefficients exceeding 0.95 for all landmarks. For bilateral measurements, preliminary analyses revealed no differences between the left and right sides in terms of growth or sex differences; thus, only the left side was included to reduce the number of comparisons. Detailed descriptions of the 3D imaging protocol and the included measurements are available at Weinberg et al. [43] and at www.facebase.org/facial_norms. Within each of the six age groups, males and females were compared on all measurements with analysis of covariance (ANCOVA). ANCOVA was used in order to compare means across the sexes while adjusting for the covariates height and age. Because of the large number of statistical tests performed and to aid interpretation, effect sizes were calculated for each comparison. For effect size, we used Cohen’s *d*, which provides a standardized way to evaluate the direction and magnitude of the difference between group means. To account for the covariate adjustment, the denominator in the standard formula for Cohen’s *d* was modified by replacing the pooled standard deviation with the square root of the adjusted within-groups mean square value from ANCOVA as a measure of pooled variation. The following guidelines were used to interpret Cohen’s *d*: 0 to 0.19 = very small; 0.20 to 0.49 = small; 0.50 to 0.79 = moderate; >0.80 = large.

**Table 2:** List of anthropometric measurements used in analysis

Measurement	Region	Landmarks involved	Collection method
Maximum cranial width	Head	Right euryon (eu_r)—left euryon (eu_l)	Spreading calipers
Minimum frontal width	Head	Right frontotemporale (ft_r)—left frontotemporale (ft_l)	Spreading calipers
Maximum facial width	Face	Right zygion (zy_r)—left zygion (zy_l)	Spreading calipers
Mandibular width	Face	Right gonion (go_r)—left gonion (go_l)	Spreading calipers
Maximum cranial length	Head	Glabella (g)—opisthocranion (op)	Spreading calipers
Cranial base width	Head	Right tragion (t_r)—left tragion (t_l)	3D Photogrammetry
Upper facial depth^a^	Face	Nasion (n)—left tragion (t_l)	3D Photogrammetry
Middle facial depth^a^	Face	Subnasale (sn)—left tragion (t_l)	3D Photogrammetry
Lower facial depth^a^	Face	Gnathion (gn)—left tragion (t_l)	3D Photogrammetry
Morphological facial height	Face	Nasion (n)—gnathion (gn)	3D Photogrammetry
Upper facial height	Face	Nasion (n)—stomion (sto)	3D Photogrammetry
Lower facial height	Face	Subnasale (sn)—gnathion (gn)	3D Photogrammetry
Intercanthal width	Eye	Right endocanthion (en_r)—left endocanthion (en_l)	3D Photogrammetry
Outercanthal width	Eye	Right exocanthion (ex_r)—left exocanthion (ex_l)	3D Photogrammetry
Palpebral fissure length^a^	Eye	Left endocanthion (en_l)—left exocanthion (ex_l)	3D Photogrammetry
Nasal width	Nose	Right alare (al_r)—left alare (al_l):	3D Photogrammetry
Subnasal width	Nose	Right subalare (sbal_r)—left subalare (sbal_l)	3D Photogrammetry
Nasal protrusion	Nose	Subnasale (sn)—pronasale (prn)	3D Photogrammetry
Nasal ala length^a^	Nose	Left alar curvature point (ac_l)—pronasale (prn)	3D Photogrammetry
Nasal height	Nose	Nasion (n)—subnasale (sn)	3D Photogrammetry
Nasal bridge length	Nose	Nasion (n)—pronasale (prn)	3D Photogrammetry
Labial fissure width	Mouth	Right chelion (ch_r)—left chelion (ch_l)	3D Photogrammetry
Philtrum width	Mouth	Right crista philtri (cph_r)—left crista philtri (cph_l)	3D Photogrammetry
Philtrum length	Mouth	Subnasale (sn)—labiale superius (ls)	3D Photogrammetry
Upper lip height	Mouth	Subnasale (sn)—stomion (sto)	3D Photogrammetry
Lower lip height	Mouth	Stomion (sto)—sublabiale (sl)	3D Photogrammetry
Upper vermilion height	Mouth	Labiale superius (ls)—stomion (sto)	3D Photogrammetry
Lower vermilion height	Mouth	Stomion (sto)—labiale inferius (li)	3D Photogrammetry
Cutaneous lower lip height	Mouth	Labiale inferius (li)—sublabiale (sl)	3D Photogrammetry

Detailed descriptions of these measurements are available at the following link: https://www.facebase.org/facial_norms/notes

^a^Indicates that only the left side of this bilateral measurement is included

Geometric morphometrics was used to investigate sex differences in facial shape [47]. Unlike traditional morphometrics, in geometric morphometrics, the coordinates (2D or 3D) associated with anatomical landmarks serve as the raw variables for analysis. The current study included the xyz coordinates corresponding to 24 standard facial soft-tissue landmarks identified on each 3D facial scan (Fig. 1). Participants were allocated to same six age categories used in the linear distance analysis. For each age group, the same analysis steps were applied. First, the raw landmark coordinates were first subjected to a Procrustes superimposition, an iterative process that scales, centers, and rotates the entire set of landmark configurations so that they are aligned within a common coordinate system [48]. The variation that remains in the relative position of landmarks reflects shape, and the newly transformed coordinates are referred to as Procrustes shape coordinates (or simply shape coordinates). The portion of shape variation due to changes in body size (allometry) and age were then removed, as these variables can affect the relationship between facial shape and sex. To accomplish this, a regression of Procrustes shape coordinates on centroid size and age was performed.

The regression residuals (size and age-adjusted shape coordinates) were then used to test for differences in mean shape between males and females. This was achieved by computing the Procrustes distance between the mean shape of each sex and calculating a permutation-based *p* value (5000 resampling runs) to determine statistical significance. The adjusted shape coordinates were then subjected to a discriminant function analysis (DFA) in order to determine the most salient aspects of facial shape for distinguishing the sexes. DFA is a multivariate data reduction technique that works by constructing a weighted variate optimized to achieve maximum separation between groups. The statistical significance of the discriminant function was determined by the Hotelling *T*^2^ test. The ability of the discriminant function to assign individuals to the correct sex (the classification accuracy) was reported and further assessed using a jackknife resampling routine, which can help provide a more realistic estimate of discriminant model performance.

Because the geometric properties of the landmarks are retained at each stage of the analysis, the shape variation associated with a given discriminant function can be modeled graphically as a change in the relative spatial positions of anatomical landmarks. This feature of geometric morphometrics allows for the creation of intuitive and meaningful visualizations of group differences in shape. In the present study, both simple 3D wireframe models and 3D surface warps were used to help visualize relevant aspects of shape variation [49].

The traditional linear distance analysis was conducted in SPSS v21 (Chicago, IL), while geometric morphometric analysis was performed using MorphoJ v1.05f [50]. The 3D surface warps were created using the program Landmark v3.0.0.6 (http://graphics.idav.ucdavis.edu/research/projects/EvoMorph).

## Results

### Linear distance analysis

Statistically significant sex differences were identified in at least one age group for 28 of the 29 traditional anthropometric measurements; only nasal height did not differ between males and females in any age group. The ANCOVA results and associated effect sizes for all age groups are summarized in Table 3; detailed results for each age group are available as supplementary material (Additional file 1: Tables S1–S6). In the early childhood group, 55 % (16/29) of the distances showed statistical evidence of sex difference, including measures of the cranial vault, nose, and eyes and measures of facial depth and width. The average effect size across the 29 measurements was 0.37, with a range of 0.01 for upper vermilion height to 1.10 for cranial base width. In the late childhood group, significant sex differences were present in 66 % (19/29) of measurements. These included the same measurements in the early childhood group, but also measures of facial height, mandibular width, and the upper lip. The average effect size was very similar to the early childhood group at 0.33, with a range of −0.01 for nasal bridge length to 0.83 for cranial base width. During puberty, the number of measurements exhibiting statistically significant sex differences dropped to 38 % (11/29), although the effect sizes were generally larger compared to earlier age groups. Sex differences were observed in many of the same measurements seen in the early and late childhood groups, with the most pronounced sex differences involving the facial depth measures. However, all of the previously seen sex differences in nasal dimensions were lost during this age span. The average effect size in the puberty group was 0.59, ranging from −0.01 for labial fissure width to 1.21 for lower facial depth.

**Table 3:** Summary of *p* values and accompanying effect sizes from ANCOVA tests comparing males and females

	Early childhood		Late childhood		Puberty		Adolescence		Young adult		Adult	
Linear distance	*p*	*d*	*p*	*d*	*p*	*d*	*p*	*d*	*p*	*d*	*p*	*d*
Maximum cranial width	*<0.001*	0.88	*<0.001*	0.73	*0.010*	0.73	*<0.001*	0.95	*<0.001*	1.32	*<0.001*	1.20
Minimum frontal width	0.738	0.10	0.203	0.14	0.353	0.37	*0.003*	0.94	0.685	0.46	*0.002*	0.80
Maximum facial width	*<0.001*	0.58	*0.001*	0.47	0.359	0.39	0.083	0.79	*<0.001*	1.67	*<0.001*	1.34
Mandibular width	*0.006*	0.47	*0.001*	0.46	0.075	0.71	*0.006*	0.78	*0.020*	0.92	*<0.001*	1.21
Maximum cranial length	*<0.001*	0.63	*<0.001*	0.82	*0.005*	0.94	*<0.001*	1.94	*0.001*	1.31	*<0.001*	1.84
Cranial base width	*<0.001*	1.10	*<0.001*	0.83	*0.001*	0.96	*<0.001*	1.41	*<0.001*	1.92	*<0.001*	1.74
Upper facial depth^a^	*<0.001*	0.68	*<0.001*	0.67	*0.001*	1.09	*<0.001*	1.65	*<0.001*	1.66	*<0.001*	1.62
Middle facial depth^a^	*<0.001*	0.68	*<0.001*	0.55	*<0.001*	1.18	*<0.001*	1.86	*<0.001*	1.90	*<0.001*	1.76
Lower facial depth^a^	*<0.001*	0.64	*0.002*	0.37	*<0.001*	1.21	*<0.001*	1.46	*<0.001*	1.97	*<0.001*	2.10
Morphological facial height	*0.026*	0.36	*0.011*	0.31	*0.030*	1.06	*0.002*	1.50	*<0.001*	1.30	*<0.001*	1.58
Upper facial height	0.098	0.26	*0.031*	0.24	0.265	0.77	*0.015*	1.37	*0.006*	0.92	*0.043*	0.81
Lower facial height	*0.015*	0.39	*0.023*	0.33	*0.001*	1.13	*<0.001*	1.41	*0.002*	1.09	*<0.001*	1.17
Intercanthal width	0.651	0.05	0.647	0.10	0.552	0.11	*0.004*	0.52	0.237	0.50	*<0.001*	0.61
Outercanthal width	*0.009*	0.37	*0.001*	0.49	*0.017*	0.63	*0.020*	0.65	0.179	0.81	*0.001*	0.94
Palpebral fissure length^a^	*0.019*	0.33	*0.002*	0.42	0.104	0.44	0.989	0.24	0.599	0.42	0.442	0.46
Nasal width	*0.025*	0.35	*0.011*	0.35	0.129	0.54	*<0.001*	1.47	*<0.001*	1.52	*<0.001*	1.57
Subnasal width	*<0.001*	0.54	*0.001*	0.46	0.922	0.10	0.321	0.50	*0.001*	0.77	*<0.001*	0.93
Nasal protrusion	0.478	−0.09	0.212	0.12	0.705	0.34	0.276	0.63	0.688	0.48	*0.040*	0.62
Nasal ala length^a^	*0.008*	0.41	*0.004*	0.35	0.084	0.78	*<0.001*	2.06	*<0.001*	2.04	*<0.001*	2.00
Nasal height	0.352	0.14	0.295	0.07	0.507	0.30	0.606	0.85	0.061	0.56	0.335	0.57
Nasal bridge length	0.257	0.17	0.737	−0.01	0.346	0.11	0.101	1.03	*0.001*	0.71	*0.019*	0.69
Labial fissure width	0.152	0.23	0.061	0.24	0.487	−0.01	*0.040*	0.36	*0.015*	0.88	*<0.001*	0.76
Philtrum width	0.090	0.28	*<0.001*	0.47	0.969	0.17	*0.044*	0.53	*<0.001*	1.05	*<0.001*	0.81
Philtrum length	*0.044*	0.30	*0.006*	0.38	*0.012*	0.78	*<0.001*	0.70	0.124	0.56	*<0.001*	0.68
Upper lip height	0.080	0.30	*0.027*	0.32	*0.004*	0.93	*<0.001*	1.19	0.098	0.64	*0.033*	0.59
Lower lip height	0.189	0.18	0.894	−0.08	0.257	0.43	*0.042*	0.67	0.018	0.53	*0.022*	0.55
Upper vermilion height	0.835	−0.01	0.450	−0.05	0.098	0.45	0.838	0.06	0.253	0.43	*0.022*	0.05
Lower vermilion height	0.135	0.23	0.921	0.02	0.603	0.19	0.574	0.17	0.417	0.06	*0.042*	0.05
Cutaneous lower lip height	0.602	0.10	0.667	−0.13	0.406	0.34	*<0.001*	0.65	*<0.001*	0.60	*<0.001*	0.64

^a^Indicates that only the left side of this bilateral measurement is included. Sign of Cohen’s *d*: positive = males larger, negative = females larger. Significant *p*-values (*p* < 0.05) are indicated in italic font

Beyond puberty, the sex differences became more pronounced, involving a wide variety of traits. In the adolescence age group, significant sex differences were present in 72 % (21/29) of measurements, involving all regions of the head and face. Both minimum frontal width and intercanthal width show evidence of dimorphism for the first time in this age group. The average effect size was 0.98, ranging from 0.06 for upper vermillion height to 2.06 for nasal ala length. For the young adult group, sex differences were observed in 66 % (19/29) of measurements. The average effect size was 1.00, ranging from 0.06 for lower vermillion height to 2.04 for nasal ala length. In the adult group, the entire craniofacial complex showed evidence of sexual dimorphism. Significant sex differences were identified in 93 % of measurements, with only palpebral fissure length and nasal height showing no statistical difference. The average effect size was 1.02, with a range of 0.05 for lower vermillion height to 2.10 for lower facial depth.

**Fig. 2:**
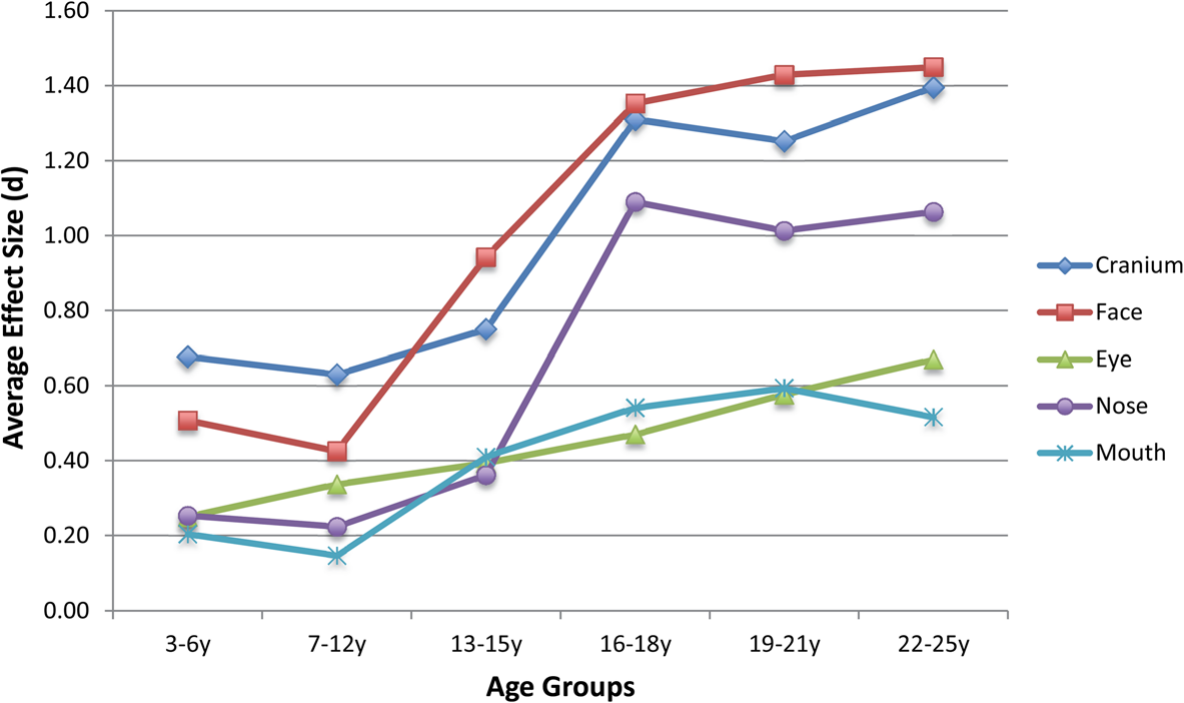
Plot of average effect size (*d*) by region for each age group. The larger the average effect size, the greater the difference between males and females. The measurements comprising each region are listed in Table 2

When measurements were grouped by anatomical region (the cranium, face, eye, nose, or mouth), different ontogenetic patterns of sexual dimorphism were apparent. Figure 2 plots the average effect size by region across the six age groups. The regions correspond to those defined in Table 2. For cranial measures, moderate dimorphism is already apparent in the early childhood group. There is a sharp increase in adolescence, after which dimorphism remains high. For facial measures, sex differences are at a low to moderate level in the early and late childhood, rising sharply at puberty and adolescence, and then leveling off by young adulthood. In contrast, eye measures show a steady linear increase in dimorphism from low levels in the early childhood to moderate levels in adults. Nose measures follow a similar pattern as cranial measures, although the level of dimorphism at each life stage is lower. These measures start out with low dimorphism, with a sharp increase at adolescence. Measures of the mouth and lips show a complex pattern, gently rising to moderate levels of dimorphism by adolescence, then showing a slight reduction in adults.

Overall patterns of craniofacial growth also differed by sex. Using the same composite anatomical regions defined above, females showed a tendency to attain adult proportions (defined as the sex-specific mean value for 22–25 year age group) earlier than males for measurements comprising the cranium, face, eye, and nose (Table 4). The sex disparity was most apparent in the nose region, where females had attained 98.3 % of their adult size by puberty compared to males at 93 %. By adolescence, males and females had both achieved over 99 % of adult nasal size. This indicates that while female nasal growth was almost complete at puberty, males continued to gain through adolescence. For mouth measurements, the same pattern of more advanced female growth was present until adolescence, at which time males had attained adult proportions while females continued to grow into adulthood.

**Table 4:** Proportion (%) of adult size attained at each age by craniofacial region

		3–6 years	7–12 years	13–15 years	16–18 years	19–21 years
Cranium	Male	88.1	93.8	97.8	99.7	98.9
	Female	90.8	96.6	100.2	99.9	100.1
Face	Male	80.3	88.1	95.2	99.0	100.1
	Female	83.7	92.3	97.3	99.2	100.4
Eye	Male	86.1	93.5	98.6	100.1	100.0
	Female	88.4	95.8	100.4	101.1	100.8
Nose	Male	73.6	83.1	93.0	99.8	99.7
	Female	77.7	88.4	98.3	99.7	100.3
Mouth	Male	78.6	85.5	94.5	100.3	100.6
	Female	81.9	90.2	95.1	97.8	99.3

22–25-year group used as the reference adult category for calculating proportions. Refer to Table 2 for which measurements comprise each craniofacial region

The following measurements showed evidence of sexual dimorphism at every age category: maximum cranial width and length, cranial base width, facial depth (upper, middle, and lower), lower facial height, and morphological facial height. Maximum facial width, mandibular width, upper facial height, outercanthal width, nasal width, nasal ala length, philtrum length, and upper lip height also showed strong evidence of dimorphism across multiple ages. Several other traits showed distinctive patterns. Palpebral fissure length showed statistical evidence of dimorphism during childhood, but these differences were subsequently lost starting at puberty. Conversely, traits such as minimum frontal width, intercanthal width, nasal protrusion, nasal bridge length, labial fissure width, and several vertical measures of lip height show little evidence of sexual dimorphism during childhood, only emerging after puberty. These data are reported in Table 3.

In terms of directionality, measurements were generally larger in males than in females at every age category (see Additional file 1: Tables S1–S6). This was likely not the result of overall sex differences in body size, as standing height was included as a covariate in all analyses. There were only a handful of exceptions, where females were larger than males. These mostly involved measures of the lips during childhood, but none of these differences were statistically significant and had very small effect sizes.

### Statistical shape analysis

A significant difference in mean facial shape between males and females was observed in every age group; the relevant Procrustes distance statistics are provided in Table 5. The trend toward increased Procrustes distance with increased age was apparent, with the exception of late childhood where there was a slight decrease. Thus, differences in shape between the average male and female face were intensified as age advanced, with a sharp increase after puberty. Differences in craniofacial size were assessed by comparing mean centroid size between the sexes. In each age group, males had significantly larger faces than females (*p* ≤ 0.002). The observed sex differences in facial shape were independent of these differences in size.

**Table 5:** Results of Procrustes distance test for differences in mean shape between males and females

Age group	Procrustes distance	*p*
Early childhood	0.01386427	0.004
Late childhood	0.01167579	0.005
Puberty	0.01494668	0.038
Adolescence	0.02472203	<0.0001
Young adult	0.02530245	<0.0001
Adult	0.03073081	<0.0001

For each age group, a single discriminant function was derived. In each case, the function was statistically significant, indicating that males and females could be distinguished on the basis of face shape. The relevant DFA statistics are presented in Table 6. In general, the resulting classification statistics revealed a similar relationship between shape discrimination and age. Looking at the cross-validation results, as age increased, the overall classification accuracy of the discriminant model tended to improve, particularly after puberty. The lowest accuracy observed was in early childhood at 67.2 %. By contrast, 91.5 % of adults could be classified as male or female correctly.

**Table 6:** Results from discriminant function analyses

				% Correctly classified		
Age group	*T* ^2^	*p*	Classification method	Male	Female	Overall
Early childhood	169.09	<0.0001	Initial assignment	84.7	85.4	85.0
			Cross-validation^a^	62.4	72.0	67.2
Late childhood	146.59	<0.0001	Initial assignment	76.4	79.1	77.8
			Cross-validation	69.1	67.3	68.2
Puberty	135.83	0.001	Initial assignment	86.3	89.1	87.7
			Cross-validation	64.7	70.9	67.8
Adolescence	226.30	<0.0001	Initial assignment	95.1	93.5	94.3
			Cross-validation	65.9	83.9	74.9
Young adult	470.09	<0.0001	Initial assignment	93.5	91.2	92.4
			Cross-validation	83.9	87.6	85.7
Adult	1250.47	<0.0001	Initial assignment	94.6	93.3	93.9
			Cross-validation	91.7	91.2	91.5

^a^Jackknife resampling used to cross-validate classification

**Fig. 3:**
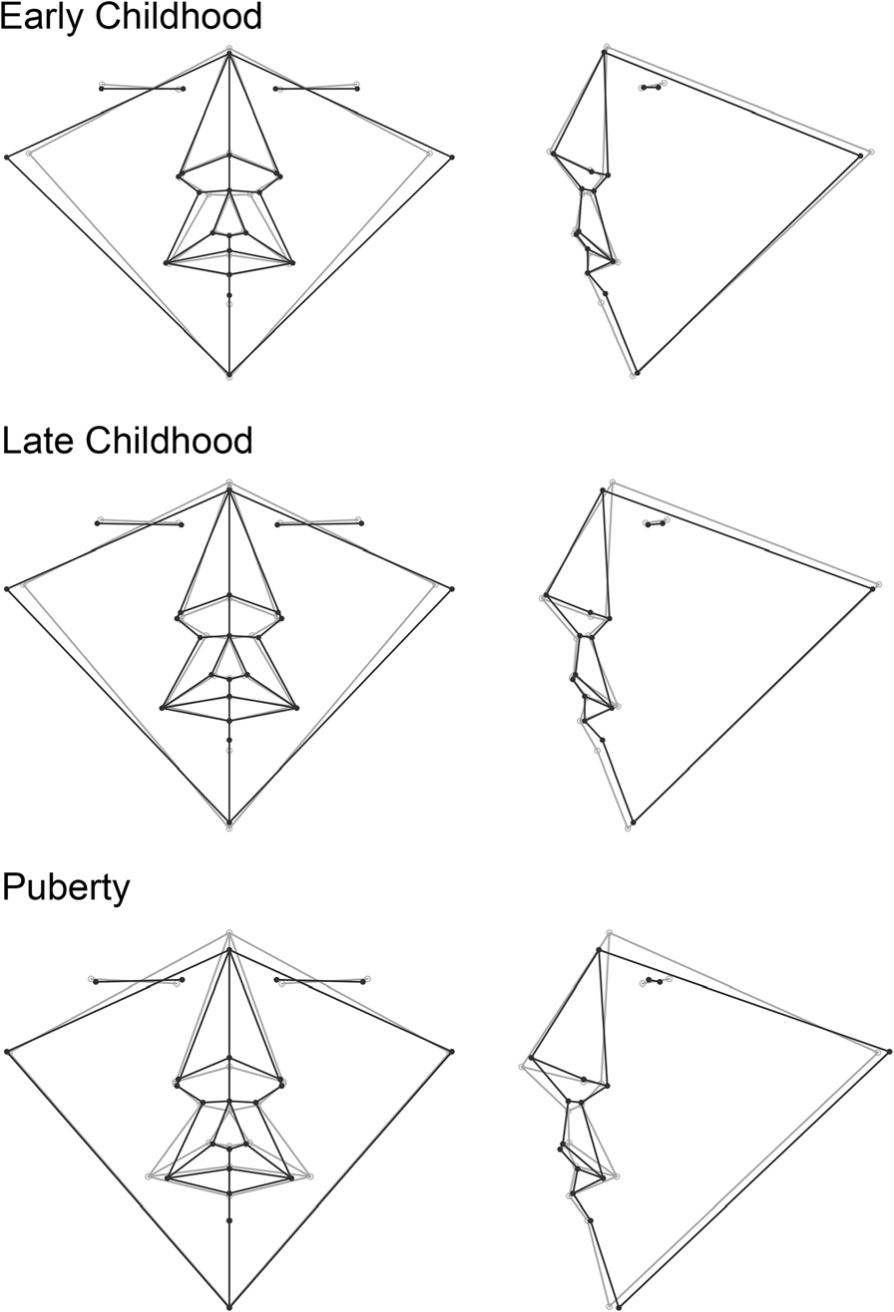
Wireframe models showing shape differences between male and female faces based on discriminant function analysis. The *black wireframe* represents the mean male face shape, while the *gray wireframe* represents the mean female shape. Each row shows results for a different age group: *top row*—early childhood group, *middle row*—late childhood, *bottom row*—puberty. The wireframes in the first column (*page right*) show the shape differences in frontal view, while the wireframes in the second column (*page left*) show the lateral or profile view. Shape changes magnified ×5 for clarity

**Fig. 4:**
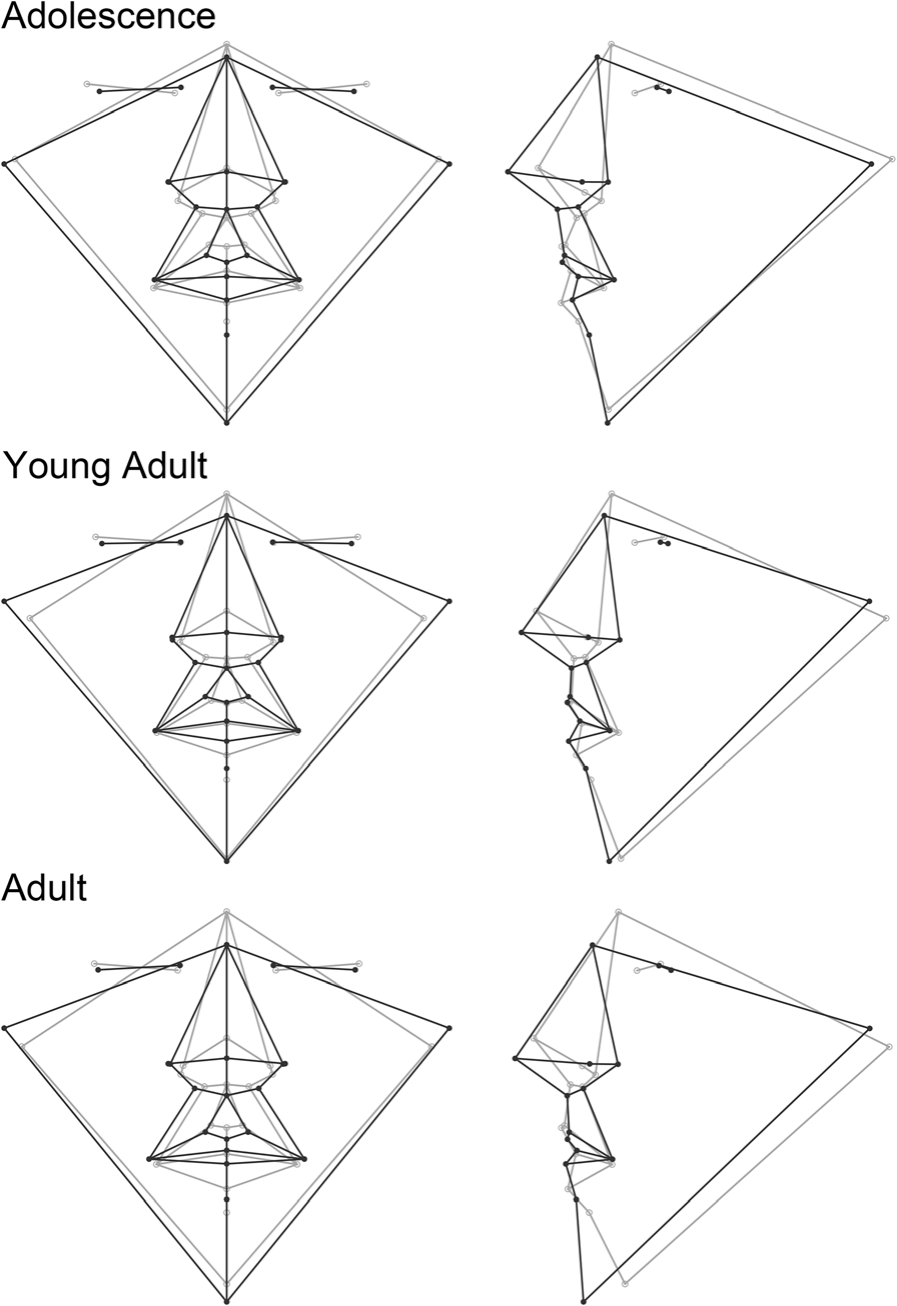
Wireframe models showing shape differences between male and female faces based on discriminant function analysis. The *black wireframe* represents the mean male face shape, while the *gray wireframe* represents the mean female shape. Each row shows results for a different age group: *top row*—adolescence, *middle row*—young adult, *bottom row*—adult. The wireframes in the first column (*page left*) show the shape differences in frontal view, while the wireframes in the second column (*page right*) show the lateral or profile view. Shape changes magnified ×5 for clarity

**Fig. 5:**
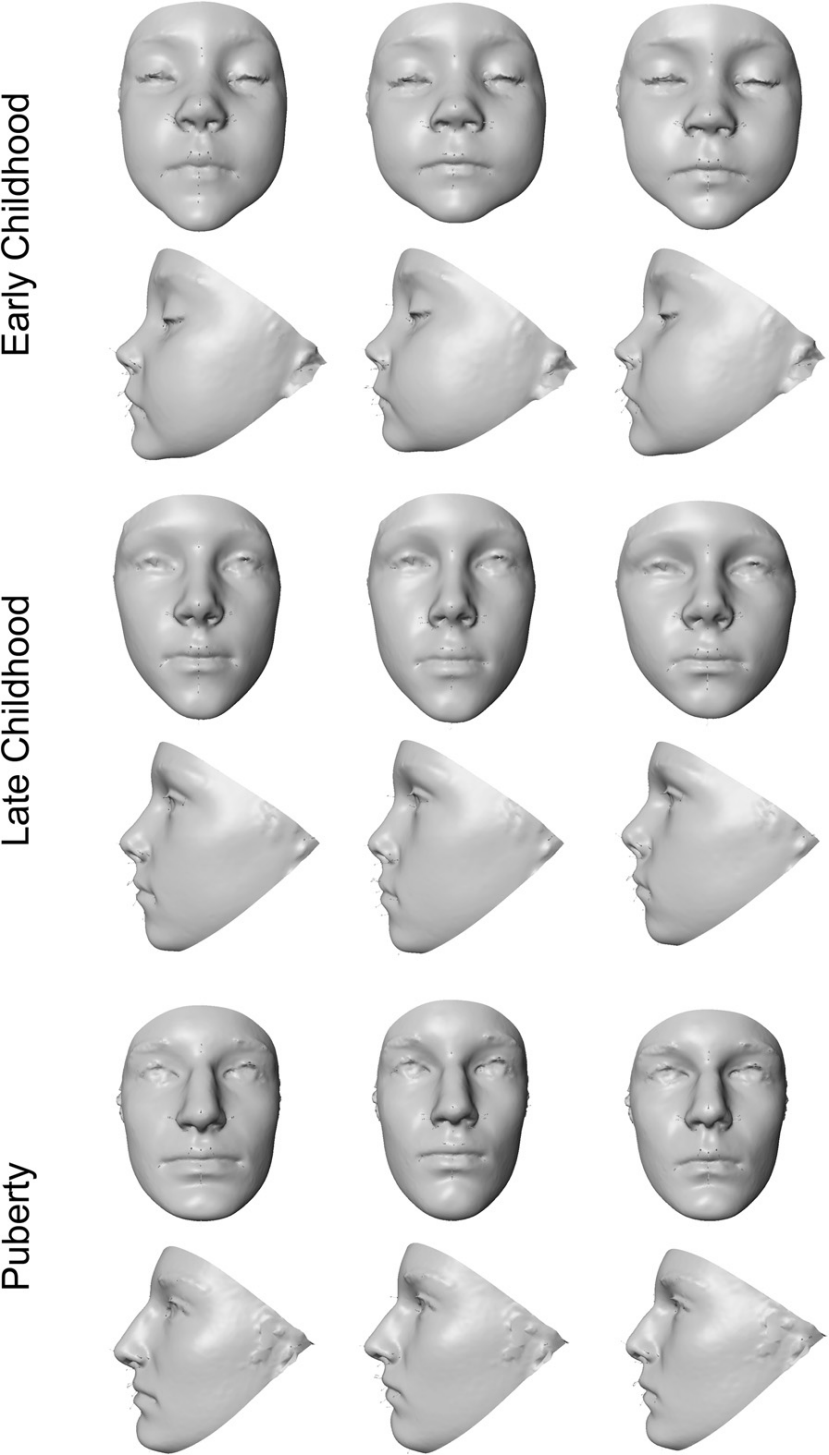
Facial surface warps showing the shape variation associated with discriminant function analysis separating males from females. Each row shows results for a different age group: *top row*—early childhood group, *middle row*—late childhood, *bottom row*—puberty. The *middle face* in each row represents an average (*gender neutral*) face for each age group. The *left face* in each row shows the average face warped to the female mean shape. The *right face* in each row shows the average face warped to the male mean shape. Shape changes magnified ×5 for clarity

**Fig. 6:**
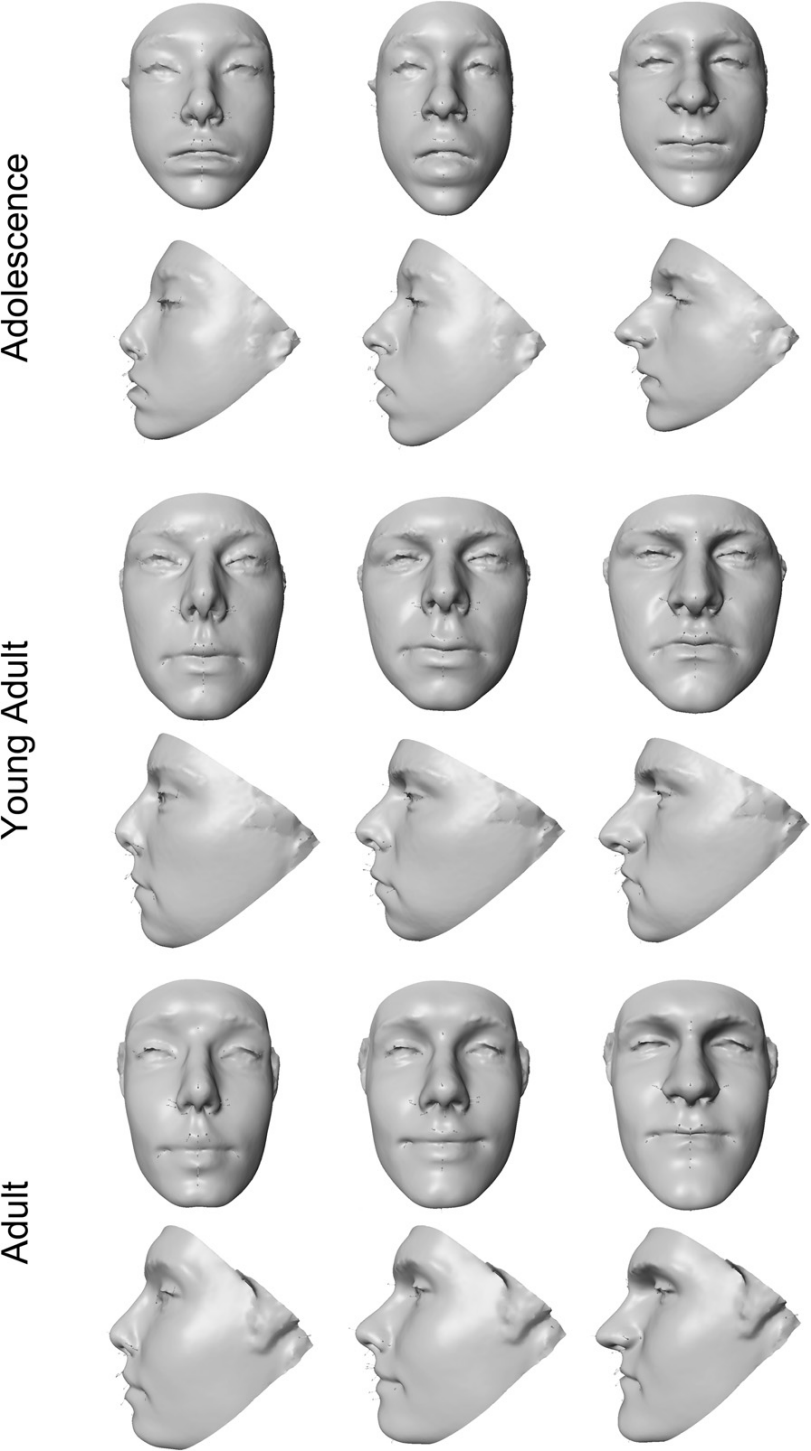
Facial surface warps showing the shape variation associated with discriminant function analysis separating males from females. Each row shows results for a different age group: *top row*—adolescence, *middle row*—young adult, *bottom row*—adult. The *middle face* in each row represents an average (*gender neutral*) face for each age group. The *left face* in each row shows the average face warped to the female mean shape. The *right face* in each row shows the average face warped to the male mean shape. Shape changes magnified ×5 for clarity

The aspects of facial shape most important for discriminating males from females are captured by the wireframe and surface models in Figs. 3, 4, 5, and 6. There were several consistent shape differences seen in multiple age groups. At every age, the inclination of the palpebral fissure was reduced (more horizontal) and the landmark nasion displaced more inferiorly and anteriorly in males. Both of these traits showed evidence increased sexual dimorphism with advancing age. Males also exhibited posterior displacement of orbital landmarks starting at puberty and most pronounced at the medial commissures (endocanthion). The position of tragion, demarking the lateral extent of the cranial base, was displaced laterally in males at all ages except puberty. In young adults and adults, this lateral displacement was accompanied by a superior and anterior shift.

There were some distinctive age-dependent changes in nasal shape between the sexes. The alare landmarks were displaced laterally in males, most apparent starting in adolescence. Pronasale, the tip of the nose, was slightly more protrusive in females through puberty. This pattern was then reversed in adolescence, with males exhibiting a much greater degree of nasal projection, as well as an inferior shift in pronasale. The combination of inferior displacement of nasion, lateral displacement of alare, and anterior and inferior displacement of pronasale in males resulted in significant sexual dimorphism in nasal shape by adulthood. This is apparent in Fig. 6, where the male nose is characterized as shorter, broader, and more projecting relative to females.

Sex differences were also apparent in the shape of the lips. Superior displacement of sublabial, the point demarcating the inferior extent of the cutaneous lower lip, was evident in males at all ages except puberty. There was also a reduction in the relative height of the vermilion portion of the lower lip, emerging at adolescence and becoming more pronounced into adulthood. By contrast, sex differences in the shape of the upper lip and philtrum were inconsistent, showing a high degree of variability across the different age groups. Finally, sex differences in the anterior position of the chin relative to the rest of the face were apparent at adolescence, with the characteristic of male facial profile emerging during this period and become more prominent into adulthood.

## Discussion

The results of this study indicate that by adulthood, most soft-tissue features of the human head and face show strong evidence of sexual dimorphism. This finding is in alignment with most published studies of craniofacial sex differences post puberty. More notable, however, are results showing evidence of numerous sex differences in pre-pubertal children. Even in the earliest age group, statistical differences were found for just over half of the anthropometric variables examined, involving all parts of the craniofacial complex. After accounting for differences in body size, 3–5 year old boys had significantly increased cranial width and length, facial width and depth, total facial height, palpebral fissure length and outercanthal width, nasal width and ala length, and philtrum length compared to girls of the same age. A handful of studies have reported evidence of similar sex differences in children this young [11, 36, 37]. One implication of these findings is that additional data will be required from even younger individuals to more precisely determine when many sexually dimorphic craniofacial features initially arise. Agnihotri and Singh [32], for example, reported that sex differences in philtrum length were detectable in newborns. Our results also challenge the conventional view that pre-pubertal sex differences are largely absent from the head and face.

While some features showed evidence of dimorphism during early childhood, other did not emerge until after puberty; these included minimum frontal width, intercanthal width, nasal protrusion, nasal bridge length, labial fissure width, and measures of lip height. When measures were grouped into regions, the magnitude (mean effect size) of the sex differences showed distinctive patterns with increased age. For cranial, facial, and nasal measurements, there was a large spike in the degree of dimorphism, occurring immediately after later childhood for facial measurements and after puberty for cranial and nasal measurements. The degree of dimorphism for all three regions then plateaued following adolescence. Eye and mouth measurements showed a very different pattern of dimorphism with increased age, with no major spike around puberty and lower levels into adulthood relative to other craniofacial regions.

These results paint a complex and heterogeneous picture of sex differences in the craniofacial complex, with the degree of dimorphism changing with age in a trait-specific manner. Some of these patterns may be explained by sex differences in craniofacial growth. It is well known that females on average achieve adult size in craniofacial features earlier than males, who experience an extended growth period [26]. This pattern is also present in the current dataset. Collapsing the measurements into composite regional units, the percentage of adult size attained for a given age group can be calculated for males and females separately. It is clear that females achieve adult size earlier than males across the craniofacial complex, which can help explain why dimorphism continues to increase into adulthood. Such differences in growth can also help explain some of the patterns observed for specific facial features, such as the large spike in dimorphism between puberty and adolescence for nasal measurements. Between the period of puberty and adolescence, male nasal growth is nearly five times that of females, who achieve adult nasal dimensions at puberty.

Geometric morphometric analysis of facial shape also revealed evidence of sexual dimorphism in every age group. Like the linear distance data, some sex differences were consistent across all age groups, while others emerged only at later stages. At all ages, the inclination of the palpebral fissures was increased in females, resulting in an upward slant compared to males. Using traditional morphometry, increased palpebral fissure inclination in females has been previously reported in adults [51], although other studies have failed to find sex differences in this trait [30, 52]. At all ages except puberty, males exhibited an increase in breadth relative to height for the midface, due primarily to the more inferior shift of nasion and more lateral displacement of the tragion points. This discrepancy may be due to individual variation in the timing of puberty, which can obscure sex differences during this period in cross-sectional samples [53]. Following puberty, the definitive adult pattern of sex differences in facial shapes was apparent, with males showing evidence of less prominent orbits, broader and more prominent noses, and greater mandibular prognathism. This constellation of features largely agrees with previous shape studies documenting sexual dimorphism in older teenagers and adults [7, 21, 34, 38]. Moreover, in another recent study by Weinberg et al. [15], many of these same traits were found to be associated with lower 2nd to 4th digit ratios in adult males—a hypothesized marker for prenatal androgen exposure. This finding is part of a growing body of evidence suggesting that adult sex differences in facial shape may be influenced by both prenatal as well as postnatal hormonal influences [54].

Our results also revealed that some of these post-puberty sex differences in shape ultimately result from a combination of changes occurring at different age points and over different spans of time. For example, the shorter, broader, and more projecting nose apparent in adult males involved at least three distinct shape changes: (1) a widening of the nose resulting from the lateral displacement of the alare landmarks at adolescence; (2) a greater nasal prominence resulting from the anterior and inferior displacement of pronasale also at adolescence; and (3) a reduction in overall nasal height resulting from the inferior and anterior displacement of nasion, starting in early childhood and becoming progressively more apparent with age. This indicates that the major craniofacial shape differences that distinguish adult males and females, even when involving a single structure, may not emerge all at once but may instead result from several distinct morphological changes occurring along different developmental trajectories.

The presence of craniofacial sexual dimorphism in young children has several important implications. The use of sex-appropriate craniofacial norms for treatment planning and outcome assessment is essential. For comparative morphological studies in general, an effort should be made to treat males and females separately and use sex-specific controls whenever possible, even when dealing with very young children. An improved understanding of how age-dependent craniofacial sex differences reflect differences in growth can also inform the way we approach therapeutic interventions. Because aspects of the face continue to grow well in past puberty in males, insults to growth at this time may have a greater impact. For females, earlier disruptions may be more damaging, since a greater proportion of craniofacial growth occurs during the pre-pubertal years.

This study demonstrates the value of large normative craniofacial datasets that both cover a wide range of ages and include a wide range of phenotypic measurements. The availability of both traditional anthropometric measures and 3D landmark coordinates in the 3D Facial Norms repository allowed for both conventional and more advanced morphometric analyses. Specifically, the use of geometric morphometrics allowed us to model aspects of shape in a comprehensive way that would be very difficult or impossible with more traditional approaches. Several limitations are also important to consider. Despite the scope and size of this study, the constructed age groups are relatively broad. Larger samples will be required to evaluate these sex differences with a greater degree of granularity and precision. It is clear for our results that many sex differences were already present in 3–5 year olds, suggesting that such differences arose even earlier in life. Thus, there is a need for future studies to include even younger individuals. Furthermore, because the 3D Facial Norms dataset is limited to US whites, the generalizability of these results to other regions and ethnicities cannot be assumed. Only the creation of large multi-ethnic normative cohorts will allow us to investigate these kinds of questions. Finally, the simple landmark-based methods used here provide limited facial coverage; for example, there were no data points included on the brow ridges or cheeks even though both areas have been shown previously to exhibit sexual dimorphism. Dense surface modeling approaches [13] can overcome some of those limitations and will be a focus of future studies.

## Conclusions

In this study we used cross-sectional data derived from the 3D Facial Norms data repository to test for sexual dimorphism of craniofacial soft-tissue morphology at different ages. To accomplish this we used both traditional morphometric methods based on linear distances and landmark-based geometric morphometrics to compare the shape of male and female faces in 1,555 individuals between 3 and 25 years of age. In our linear distance data, we found sex differences in every age group in multiple aspects of the craniofacial complex. Craniofacial sex differences generally increased with age, with large spikes in nasal, cranial and facial dimorphism emerging after puberty. We also observed sex differences in facial shape at each age, with some dimorphic features already present in the youngest age group and persisting into adulthood. Other dimorphic features, primarily involving the nose and chin, only emerged after puberty. Our findings show that soft-tissue facial sex differences do not emerge all at once following puberty, but arise in a temporally heterogeneous and trait-specific manner during ontogeny.

## Availability of data

All of the data used in this paper is part of the 3D Facial Norms Project and is freely available through the FaceBase Consortium (www.facebase.org). These data include craniofacial measurements, demographic descriptors, 3D landmark coordinates, and the raw 3D facial surfaces from every subject. Interested investigators are encouraged to apply for access.

## Additional file

Additional file 1: A supplement to this article contains tables of detailed descriptive statistics and ANCOVA results for each age group (Tables S1–S6) as well as plots of unadjusted means and associated 95 % confidence intervals for each linear distance as a function of sex and age (Figures S1–S29).
