# Supplementary material for: Using the 3D Facial Norms Database to investigate craniofacial sexual dimorphism in healthy children, adolescents, and adults
Source: Biol Sex Differ. 2016 Apr 22;7:23. doi: 10.1186/s13293-016-0076-8 (PMC4841054; doi:10.1186/s13293-016-0076-8)
Supplement: Additional file 1: — A supplement to this article contains tables of detailed descriptive statistics and ANCOVA results for each age group (Tables S1–S6) as well as plots of unadjusted means and associated 95 % confidence intervals for each linear distance as a function of sex and age (Figures S1–S29). [file 13293_2016_76_MOESM1_ESM.docx]

Additional file 1

**Supplemental Material for *Using the 3D Facial Norms Database to Investigate Craniofacial Sexual Dimorphism in Healthy Children, Adolescents, and Adults* by Kesterke et al.**

List of supplementary tables:

**Table S1**. Descriptive statistics and ANCOVA results for the early childhood age group.

**Table S2**. Descriptive statistics and ANCOVA results for the late childhood age group.

**Table S3**. Descriptive statistics and ANCOVA results for the puberty age group.

**Table S4**. Descriptive statistics and ANCOVA results for the adolescence age group.

**Table S5**. Descriptive statistics and ANCOVA results for the young adult age group.

**Table S6**. Descriptive statistics and ANCOVA results for the adult age group.

List of supplementary figures:

**Figures S1 – S29** show plots of unadjusted means and associated 95% confidence intervals for each linear distance as a function of sex and age.

**Table S1**. Descriptive statistics and detailed ANCOVA results for the early childhood age group.

|  | Males | | | Females | | | Statistics | | | |
| --- | --- | --- | --- | --- | --- | --- | --- | --- | --- | --- |
| Variable | N | Mean | SD | N | Mean | SD | Covariates ^b^ | p | d | Mean diff (95%CI) |
| Maximum cranial width | 91 | 142.14 | 5.14 | 89 | 137.62 | 5.04 |  | **< 0.001** | 0.88 | 4.44 (2.95:5.92) |
| Minimum frontal width | 92 | 93.45 | 7.04 | 90 | 92.87 | 8.21 | Age* | 0.738 | 0.10 | 0.36 (-1.74:2.46) |
| Maximum facial width | 91 | 117.01 | 5.94 | 89 | 113.20 | 6.31 | Height** | **< 0.001** | 0.58 | 3.58 (1.89:5.26) |
| Mandibular width | 91 | 83.32 | 5.86 | 89 | 80.98 | 5.64 | Height*** | **0.006** | 0.47 | 2.12 (0.62:3.61) |
| Maximum cranial length | 90 | 176.40 | 6.98 | 90 | 172.30 | 6.76 | Height*** | **< 0.001** | 0.63 | 3.86 (1.97:5.74) |
| Cranial base width | 93 | 127.57 | 4.49 | 87 | 123.00 | 4.63 | Height*** | **< 0.001** | 1.10 | 4.35 (3.16:5.53) |
| Upper facial depth ^a^ | 93 | 105.58 | 5.12 | 88 | 102.63 | 4.47 | Height*** | **< 0.001** | 0.68 | 2.68 (1.49:3.87) |
| Middle facial depth ^a^ | 93 | 106.90 | 5.10 | 88 | 103.95 | 4.72 | Height*** | **< 0.001** | 0.68 | 2.65 (1.47:3.84) |
| Lower facial depth ^a^ | 87 | 114.95 | 5.89 | 84 | 112.01 | 5.65 | Height*** | **< 0.001** | 0.64 | 2.59 (1.27:3.91) |
| Morphological facial height | 89 | 98.02 | 6.89 | 89 | 96.06 | 5.46 | Height***, Age* | **0.026** | 0.36 | 1.75 (0.21:3.29) |
| Upper facial height | 95 | 60.78 | 4.94 | 93 | 59.70 | 3.73 | Height***, Age* | 0.098 | 0.26 | 0.91 (-0.17:1.98) |
| Lower facial height | 89 | 57.56 | 4.58 | 89 | 55.98 | 3.97 | Height** | **0.015** | 0.39 | 1.47 (0.29:2.66) |
| Intercanthal width | 94 | 28.96 | 2.33 | 92 | 28.77 | 2.35 | Height** | 0.651 | 0.05 | 0.15 (-0.50:0.80) |
| Outercanthal width | 92 | 76.29 | 3.49 | 91 | 74.99 | 3.66 | Height*** | **0.009** | 0.37 | 1.23 (0.31:2.16) |
| Palpebral fissure length ^a^ | 92 | 24.25 | 1.49 | 92 | 23.68 | 1.80 |  | **0.019** | 0.33 | 0.55 (0.09:1.01) |
| Nasal width | 95 | 27.97 | 1.99 | 92 | 27.32 | 1.78 | Height** | **0.025** | 0.35 | 0.60 (0.08:1.12) |
| Subnasal width | 94 | 15.80 | 2.07 | 93 | 14.58 | 2.49 | Height** | **< 0.001** | 0.54 | 1.18 (0.53:1.83) |
| Nasal protrusion | 95 | 14.62 | 1.33 | 93 | 14.70 | 1.39 | Height**, Age* | 0.478 | -0.09 | -0.13 (-0.48:0.23) |
| Nasal ala length ^a^ | 95 | 23.30 | 1.46 | 93 | 22.75 | 1.53 | Height***, Age* | **0.008** | 0.41 | 0.49 (0.13:0.86) |
| Nasal height | 95 | 43.08 | 4.24 | 93 | 42.52 | 3.19 | Height*, Age** | 0.352 | 0.14 | 0.45 (-0.50:1.39) |
| Nasal bridge length | 95 | 35.00 | 3.96 | 93 | 34.38 | 2.90 | Height*, Age* | 0.257 | 0.17 | 0.52 (-0.38:1.42) |
| Labial fissure width | 95 | 37.06 | 3.34 | 93 | 36.28 | 3.69 |  | 0.152 | 0.23 | 0.72 (-0.27:1.70) |
| Philtrum width | 95 | 9.60 | 1.65 | 93 | 9.22 | 1.23 | Height**, Age** | 0.090 | 0.28 | 0.36 (-0.06:0.77) |
| Philtrum length | 95 | 13.35 | 2.07 | 93 | 12.74 | 1.83 | Height*** | **0.044** | 0.30 | 0.56 (0.01:1.11) |
| Upper lip height | 95 | 18.25 | 1.97 | 93 | 17.74 | 1.79 | Height*** | 0.080 | 0.30 | 0.46 (-0.06:0.98) |
| Lower lip height | 95 | 14.98 | 2.52 | 92 | 14.54 | 1.95 |  | 0.189 | 0.18 | 0.44 (-0.22:1.09) |
| Upper vermilion height | 95 | 6.47 | 1.68 | 93 | 6.51 | 1.34 |  | 0.835 | -0.01 | -0.05 (-0.49:0.39) |
| Lower vermilion height | 95 | 6.91 | 2.65 | 92 | 6.39 | 2.14 |  | 0.135 | 0.23 | 0.53 (-0.17:1.23) |
| Cutaneous lower lip height | 95 | 9.06 | 1.83 | 92 | 8.91 | 1.84 |  | 0.602 | 0.10 | 0.14 (-0.93:0.67) |

^a^ only left variable included. ^b^ Significance of covariates indicated by * ≤ 0.05, ** ≤ 0.01, *** ≤ 0.001. d = Cohen’s d effect size measure (see text for details). Mean diff = signed difference in mm between covariate-adjusted male and female means. All mean and SD values in mm.

**Table S2**. Descriptive statistics and detailed ANCOVA results for the late childhood age group.

|  | Males | | | Females | | | Statistics | | | |
| --- | --- | --- | --- | --- | --- | --- | --- | --- | --- | --- |
| Variable | N | Mean | SD | N | Mean | SD | Covariates ^b^ | p | d | Mean diff (95%CI) |
| Maximum cranial width | 111 | 146.97 | 6.15 | 99 | 142.62 | 6.25 | Height***, Age* | **<0.001** | 0.73 | 4.12 (2.54:5.71) |
| Minimum frontal width | 111 | 104.21 | 8.72 | 99 | 103.07 | 8.81 |  | 0.203 | 0.14 | 1.47 (-0.80:3.74) |
| Maximum facial width | 111 | 125.87 | 7.81 | 96 | 123.04 | 6.78 | Height*** | **0.001** | 0.47 | 2.85 (1.17:4.52) |
| Mandibular width | 106 | 92.37 | 7.04 | 92 | 89.89 | 6.77 | Height*** | **0.001** | 0.46 | 2.62 (1.04:4.20) |
| Maximum cranial length | 108 | 183.57 | 8.71 | 96 | 178.11 | 6.90 | Height*** | **<0.001** | 0.82 | 5.45 (3.57:7.32) |
| Cranial base width | 121 | 137.34 | 6.77 | 113 | 133.01 | 6.66 | Height*** | **<0.001** | 0.83 | 4.43 (3.04:5.81) |
| Upper facial depth ^a^ | 121 | 113.95 | 6.19 | 114 | 110.98 | 5.14 | Height*** | **<0.001** | 0.67 | 3.05 (1.89:4.21) |
| Middle facial depth ^a^ | 121 | 117.60 | 6.35 | 114 | 115.25 | 5.66 | Height***, Age* | **<0.001** | 0.55 | 2.54 (1.43:3.65) |
| Lower facial depth ^a^ | 121 | 128.32 | 8.21 | 112 | 126.47 | 7.88 | Height***, Age* | **0.002** | 0.37 | 2.20 (0.83:3.58) |
| Morphological facial height | 124 | 108.18 | 7.24 | 113 | 106.70 | 6.80 | Height***, Age* | **0.011** | 0.31 | 1.72 (0.40:3.05) |
| Upper facial height | 124 | 67.42 | 5.46 | 115 | 66.47 | 4.42 | Height***, Age** | **0.031** | 0.24 | 1.13 (0.10:2.15) |
| Lower facial height | 124 | 62.31 | 4.69 | 113 | 61.06 | 4.75 | Height*** | **0.023** | 0.33 | 1.24 (0.17:2.31) |
| Intercanthal width | 124 | 31.25 | 2.89 | 115 | 31.08 | 2.57 | Height*** | 0.647 | 0.10 | 0.14 (-0.51:0.79) |
| Outercanthal width | 121 | 83.04 | 4.56 | 111 | 81.29 | 4.17 | Height*** | **0.001** | 0.49 | 1.67 (0.69:2.65) |
| Palpebral fissure length ^a^ | 121 | 26.43 | 1.92 | 111 | 25.68 | 1.79 | Height*** | **0.002** | 0.42 | 0.72 (0.26:1.19) |
| Nasal width | 124 | 30.75 | 2.41 | 115 | 30.07 | 2.64 | Height*** | **0.011** | 0.35 | 0.69 (0.16:1.23) |
| Subnasal width | 124 | 16.96 | 2.15 | 115 | 15.99 | 2.14 |  | **0.001** | 0.46 | 0.97 (0.42:1.51) |
| Nasal protrusion | 124 | 17.02 | 1.93 | 115 | 16.87 | 1.80 | Height***, Age*** | 0.212 | 0.12 | 0.24 (-0.14:0.61) |
| Nasal ala length ^a^ | 124 | 27.05 | 2.40 | 115 | 26.53 | 2.26 | Height***, Age*** | **0.004** | 0.35 | 0.61 (0.19:1.02) |
| Nasal height | 124 | 48.90 | 4.72 | 115 | 48.63 | 3.87 | Height***, Age*** | 0.295 | 0.07 | 0.48 (-0.42:1.39) |
| Nasal bridge length | 124 | 40.43 | 4.31 | 115 | 40.47 | 3.74 | Height***, Age*** | 0.737 | -0.01 | 0.15 (-0.71:1.00) |
| Labial fissure width | 123 | 42.13 | 4.70 | 115 | 41.22 | 3.89 | Height*** | 0.061 | 0.24 | 0.97 (-0.05:1.98) |
| Philtrum width | 124 | 10.69 | 1.55 | 115 | 10.04 | 1.55 | Height* | **<0.001** | 0.47 | 0.68 (0.31:1.06) |
| Philtrum length | 124 | 13.71 | 2.03 | 115 | 12.95 | 2.07 |  | **0.006** | 0.38 | 0.74 (0.21:1.27) |
| Upper lip height | 124 | 19.31 | 2.19 | 115 | 18.67 | 2.17 | Height*** | **0.027** | 0.32 | 0.61 (0.07:1.15) |
| Lower lip height | 124 | 16.08 | 2.34 | 115 | 16.18 | 2.01 |  | 0.894 | -0.08 | -0.04 (-0.59:0.52) |
| Upper vermilion height | 124 | 7.14 | 1.51 | 115 | 7.26 | 1.48 | Height*** | 0.450 | -0.05 | -0.14 (-0.50:0.22) |
| Lower vermilion height | 124 | 7.87 | 2.41 | 115 | 7.90 | 1.73 |  | 0.921 | 0.02 | 0.03 (-0.51:0.56) |
| Cutaneous lower lip height | 124 | 9.60 | 1.88 | 115 | 9.67 | 1.89 |  | 0.667 | -0.13 | -0.11 (-0.59:0.38) |

^a^ only left variable included. ^b^ Significance of covariates indicated by * ≤ 0.05, ** ≤ 0.01, *** ≤ 0.001. d = Cohen’s d effect size measure (see text for details). Mean diff = signed difference in mm between covariate-adjusted male and female means. All mean and SD values in mm.

**Table S3**. Descriptive statistics and detailed ANCOVA results for the puberty age group.

|  | Males | | | Females | | | Statistics | | | |
| --- | --- | --- | --- | --- | --- | --- | --- | --- | --- | --- |
| Variable | N | Mean | SD | N | Mean | SD | Covariates ^b^ | p | d | Mean diff (95%CI) |
| Maximum cranial width | 47 | 150.92 | 5.56 | 53 | 147.06 | 5.07 |  | **0.010** | 0.73 | 3.10 (0.76:5.44) |
| Minimum frontal width | 47 | 109.43 | 7.88 | 53 | 106.57 | 7.73 |  | 0.353 | 0.37 | 1.60 (-1.80:5.00) |
| Maximum facial width | 46 | 131.83 | 6.89 | 53 | 129.25 | 5.40 | Height* | 0.359 | 0.39 | 1.24 (-1.43:3.90) |
| Mandibular width | 45 | 98.84 | 6.09 | 50 | 94.72 | 5.86 | Height*** | 0.075 | 0.71 | 2.31 (-0.24:4.85) |
| Maximum cranial length | 46 | 191.76 | 8.06 | 53 | 185.57 | 5.67 | Height** | **0.005** | 0.94 | 4.26 (1.33:7.19) |
| Cranial base width | 50 | 143.75 | 6.50 | 55 | 138.27 | 4.74 | Height** | **0.001** | 0.96 | 4.05 (1.72:6.38) |
| Upper facial depth ^a^ | 50 | 120.82 | 4.84 | 55 | 116.23 | 3.98 | Height*** | **0.001** | 1.09 | 3.09 (1.32:4.85) |
| Middle facial depth ^a^ | 50 | 126.13 | 4.98 | 55 | 120.91 | 4.19 | Height*** | **<0.001** | 1.18 | 3.75 (1.89:5.60) |
| Lower facial depth ^a^ | 50 | 141.20 | 6.97 | 55 | 133.99 | 5.61 | Height*** | **<0.001** | 1.21 | 4.74 (2.28:7.20) |
| Morphological facial height | 51 | 119.73 | 7.06 | 56 | 113.60 | 6.78 | Height*** | **0.030** | 1.06 | 2.72 (0.27:5.18) |
| Upper facial height | 51 | 74.09 | 4.37 | 56 | 71.28 | 4.31 | Height*** | 0.265 | 0.77 | 0.90 (-0.69:2.50) |
| Lower facial height | 51 | 68.62 | 5.34 | 56 | 63.29 | 5.11 | Height*** | **0.001** | 1.13 | 3.30 (1.30:5.30) |
| Intercanthal width | 51 | 32.01 | 2.95 | 56 | 31.65 | 2.31 |  | 0.552 | 0.11 | 0.34 (-0.78:1.46) |
| Outercanthal width | 49 | 87.87 | 3.96 | 56 | 85.44 | 3.62 |  | **0.017** | 0.63 | 1.98 (0.36:3.61) |
| Palpebral fissure length ^a^ | 49 | 28.59 | 2.15 | 56 | 27.65 | 2.10 |  | 0.104 | 0.44 | 0.76 (-0.16:1.67) |
| Nasal width | 51 | 34.10 | 2.96 | 56 | 32.75 | 2.11 | Height* | 0.129 | 0.54 | 0.81 (-0.24:1.86) |
| Subnasal width | 51 | 17.81 | 2.39 | 56 | 17.52 | 2.23 |  | 0.922 | 0.10 | 0.05 (-0.92:1.01) |
| Nasal protrusion | 51 | 19.59 | 1.79 | 56 | 19.03 | 1.82 | Height*** | 0.705 | 0.34 | -0.13 (-0.81:0.55) |
| Nasal ala length ^a^ | 51 | 31.78 | 2.60 | 56 | 30.29 | 1.83 | Height***, Age* | 0.084 | 0.78 | 0.74 (-0.10:1.58) |
| Nasal height | 51 | 54.13 | 3.59 | 56 | 53.20 | 3.76 | Height*** | 0.507 | 0.30 | -0.47 (-1.87:0.93) |
| Nasal bridge length | 51 | 45.92 | 3.36 | 56 | 45.63 | 3.57 | Height** | 0.346 | 0.11 | -0.66 (-2.04:0.72) |
| Labial fissure width | 51 | 45.58 | 4.61 | 56 | 45.66 | 3.74 |  | 0.487 | -0.01 | -0.62 (-2.38:1.14) |
| Philtrum width | 51 | 11.94 | 1.72 | 56 | 11.63 | 1.69 |  | 0.969 | 0.17 | 0.01 (-0.70:0.73) |
| Philtrum length | 51 | 14.50 | 2.72 | 56 | 12.71 | 2.08 | Height* | **0.012** | 0.78 | 1.28 (0.28:2.27) |
| Upper lip height | 51 | 21.02 | 2.39 | 56 | 19.03 | 2.13 | Height*** | **0.004** | 0.93 | 1.36 (0.45:2.27) |
| Lower lip height | 51 | 17.80 | 2.79 | 56 | 16.81 | 2.33 |  | 0.257 | 0.43 | 0.62 (-0.46:1.69) |
| Upper vermilion height | 51 | 8.17 | 1.63 | 56 | 7.45 | 1.44 |  | 0.098 | 0.45 | 0.55 (-0.10:1.19) |
| Lower vermilion height | 51 | 9.09 | 1.92 | 56 | 8.73 | 2.10 |  | 0.603 | 0.19 | 0.23 (-0.63:1.08) |
| Cutaneous lower lip height | 51 | 10.44 | 2.56 | 56 | 9.78 | 1.88 |  | 0.406 | 0.34 | 0.40 (-0.54:1.34) |

^a^ only left variable included. ^b^ Significance of covariates indicated by * ≤ 0.05, ** ≤ 0.01, *** ≤ 0.001. d = Cohen’s d effect size measure (see text for details). Mean diff = signed difference in mm between covariate-adjusted male and female means. All mean and SD values in mm.

**Table S4**. Descriptive statistics and detailed ANCOVA results for the adolescence age group.

|  | Males | | | Females | | | Statistics | | | |
| --- | --- | --- | --- | --- | --- | --- | --- | --- | --- | --- |
| Variable | N | Mean | SD | N | Mean | SD | Covariates ^b^ | p | d | Mean diff (95%CI) |
| Maximum cranial width | 44 | 152.43 | 7.08 | 53 | 146.51 | 5.41 |  | **<0.001** | 0.95 | 6.31 (2.85:9.77) |
| Minimum frontal width | 44 | 110.57 | 6.32 | 53 | 104.40 | 6.69 |  | **0.003** | 0.94 | 5.66 (2.04:9.29) |
| Maximum facial width | 38 | 135.03 | 6.76 | 47 | 130.55 | 4.64 |  | 0.083 | 0.79 | 2.98 (-0.40:6.35) |
| Mandibular width | 44 | 100.96 | 6.46 | 53 | 95.79 | 6.63 |  | **0.006** | 0.78 | 5.13 (1.47:8.78) |
| Maximum cranial length | 43 | 198.81 | 6.65 | 53 | 187.08 | 5.80 | Height* | **<0.001** | 1.94 | 9.27 (5.91:12.62) |
| Cranial base width | 44 | 146.82 | 5.37 | 64 | 139.22 | 5.36 |  | **<0.001** | 1.41 | 7.75 (5.00:10.50) |
| Upper facial depth ^a^ | 45 | 125.51 | 5.07 | 65 | 118.23 | 4.00 |  | **<0.001** | 1.65 | 6.00 (3.77:8.22) |
| Middle facial depth ^a^ | 45 | 131.58 | 5.28 | 65 | 123.43 | 3.86 | Height* | **<0.001** | 1.86 | 6.87 (4.66:9.07) |
| Lower facial depth ^a^ | 43 | 146.74 | 6.99 | 64 | 138.30 | 5.41 | Height*** | **<0.001** | 1.46 | 5.48 (2.56:8.41) |
| Morphological facial height | 44 | 125.52 | 8.04 | 64 | 116.26 | 5.46 | Height*** | **0.002** | 1.50 | 5.03 (1.90:8.15) |
| Upper facial height | 46 | 77.69 | 5.50 | 65 | 71.82 | 3.91 | Height*** | **0.015** | 1.37 | 2.68 (0.53:4.84) |
| Lower facial height | 44 | 72.24 | 5.58 | 64 | 65.02 | 4.86 | Height* | **<0.001** | 1.41 | 5.53 (2.94:8.11) |
| Intercanthal width | 46 | 32.90 | 2.71 | 65 | 31.42 | 2.97 |  | **0.004** | 0.52 | 2.41 (0.70:3.58) |
| Outercanthal width | 45 | 89.10 | 4.54 | 65 | 86.37 | 3.99 |  | **0.020** | 0.65 | 2.52 (0.40:4.63) |
| Palpebral fissure length ^a^ | 45 | 28.66 | 2.01 | 65 | 28.16 | 2.17 |  | 0.989 | 0.24 | -0.01 (-1.05:1.04) |
| Nasal width | 46 | 36.32 | 2.61 | 65 | 32.84 | 2.46 | Height**, Age** | **<0.001** | 1.47 | 2.60 (1.41:3.79) |
| Subnasal width | 46 | 18.69 | 2.61 | 64 | 17.53 | 2.18 |  | 0.321 | 0.50 | 0.60 (-0.59:1.78) |
| Nasal protrusion | 46 | 21.17 | 2.37 | 65 | 19.73 | 2.35 | Height* | 0.276 | 0.63 | 0.63 (-0.51:1.78) |
| Nasal ala length ^a^ | 46 | 35.22 | 2.52 | 65 | 31.19 | 1.91 | Height***, Age*** | **<0.001** | 2.06 | 3.17 (2.18:4.15) |
| Nasal height | 46 | 56.92 | 4.45 | 65 | 53.90 | 3.41 | Height*** | 0.606 | 0.85 | 0.47 (-1.33:2.27) |
| Nasal bridge length | 46 | 49.74 | 4.33 | 65 | 46.01 | 3.43 | Height*** | 0.101 | 1.03 | 1.52 (-0.30:3.34) |
| Labial fissure width | 46 | 48.59 | 4.42 | 65 | 47.17 | 3.57 |  | **0.040** | 0.36 | 2.07 (0.10:4.03) |
| Philtrum width | 46 | 12.98 | 1.99 | 65 | 11.99 | 1.88 | Age** | **0.044** | 0.53 | 0.97 (0.03:1.91) |
| Philtrum length | 46 | 15.59 | 2.64 | 65 | 12.41 | 2.52 |  | **<0.001** | 0.70 | 2.97 (1.67:4.26) |
| Upper lip height | 46 | 22.05 | 2.81 | 65 | 18.79 | 2.72 |  | **<0.001** | 1.19 | 2.74 (1.36:4.13) |
| Lower lip height | 44 | 19.39 | 2.07 | 65 | 17.64 | 2.25 |  | **0.042** | 0.67 | 1.37 (0.05:2.70) |
| Upper vermilion height | 46 | 7.77 | 1.75 | 65 | 7.68 | 1.45 |  | 0.838 | 0.06 | -0.08 (-0.89:0.72) |
| Lower vermilion height | 46 | 9.92 | 2.18 | 65 | 9.57 | 1.99 |  | 0.574 | 0.17 | -0.29 (-1.33:0.74) |
| Cutaneous lower lip height | 44 | 11.52 | 2.72 | 65 | 9.95 | 2.16 |  | **<0.001** | 0.65 | 1.61 (0.38:2.83) |

^a^ only left variable included. ^b^ Significance of covariates indicated by * ≤ 0.05, ** ≤ 0.01, *** ≤ 0.001. d = Cohen’s d effect size measure (see text for details). Mean diff = signed difference in mm between covariate-adjusted male and female means. All mean and SD values in mm.

**Table S5**. Descriptive statistics and detailed ANCOVA results for the young adult age group.

|  | Males | | | Females | | | Statistics | | | |
| --- | --- | --- | --- | --- | --- | --- | --- | --- | --- | --- |
| Variable | N | Mean | SD | N | Mean | SD | Covariates ^b^ | p | d | Mean diff (95%CI) |
| Maximum cranial width | 96 | 153.48 | 5.54 | 185 | 147.14 | 4.43 | Height** | **<0.001** | 1.32 | 4.70 (3.02:6.37) |
| Minimum frontal width | 96 | 106.67 | 5.93 | 185 | 103.79 | 6.58 | Height***, Age* | 0.685 | 0.46 | 0.45 (-1.73:2.63) |
| Maximum facial width | 92 | 138.01 | 4.75 | 173 | 130.29 | 4.77 | Height*** | **<0.001** | 1.67 | 5.08 (3.41:6.75) |
| Mandibular width | 96 | 102.89 | 7.79 | 183 | 96.64 | 6.44 | Height*** | **0.020** | 0.92 | 2.82 (0.45:5.19) |
| Maximum cranial length | 96 | 196.77 | 6.63 | 185 | 188.93 | 6.08 | Height*** | **0.001** | 1.31 | 3.66 (1.57:5.76) |
| Cranial base width | 96 | 147.86 | 4.69 | 204 | 139.04 | 4.90 | Height*** | **<0.001** | 1.92 | 5.71 (4.13:7.28) |
| Upper facial depth ^a^ | 96 | 126.41 | 4.69 | 207 | 119.24 | 4.46 | Height*** | **<0.001** | 1.66 | 4.19 (2.71:5.67) |
| Middle facial depth ^a^ | 96 | 132.54 | 4.70 | 207 | 124.28 | 4.50 | Height*** | **<0.001** | 1.90 | 5.27 (3.78:6.75) |
| Lower facial depth ^a^ | 94 | 149.89 | 6.04 | 202 | 138.61 | 6.24 | Height*** | **<0.001** | 1.97 | 6.29 (4.31:8.26) |
| Morphological facial height | 95 | 126.44 | 6.24 | 210 | 118.78 | 6.11 | Height*** | **<0.001** | 1.30 | 3.87 (1.85:5.89) |
| Upper facial height | 98 | 77.42 | 4.17 | 215 | 73.74 | 4.01 | Height*** | **0.006** | 0.92 | 1.87 (0.53:3.21) |
| Lower facial height | 95 | 72.64 | 5.55 | 210 | 67.04 | 5.19 | Height*** | **0.002** | 1.09 | 2.87 (1.10:4.63) |
| Intercanthal width | 98 | 32.44 | 2.78 | 215 | 31.17 | 2.49 | Height* | 0.237 | 0.50 | 0.52 (-0.34:1.38) |
| Outercanthal width | 98 | 89.10 | 4.10 | 211 | 86.18 | 3.57 | Height*** | 0.179 | 0.81 | 0.84 (-0.39:2.06) |
| Palpebral fissure length ^a^ | 98 | 29.02 | 2.012 | 212 | 28.19 | 2.00 | Height** | 0.599 | 0.42 | 0.18 (-0.49:0.85) |
| Nasal width | 98 | 35.90 | 2.44 | 215 | 32.46 | 2.22 | Height*** | **<0.001** | 1.52 | 2.56 (1.80:3.33) |
| Subnasal width | 98 | 18.86 | 2.41 | 215 | 17.07 | 2.31 |  | **0.001** | 0.77 | 1.33 (0.54:2.11) |
| Nasal protrusion | 98 | 21.24 | 2.36 | 215 | 20.25 | 1.96 | Height*** | 0.688 | 0.48 | 0.14 (-0.55:0.84) |
| Nasal ala length ^a^ | 98 | 35.30 | 2.35 | 214 | 31.47 | 1.71 | Height*** | **<0.001** | 2.04 | 2.86 (2.23:3.49) |
| Nasal height | 98 | 56.70 | 3.73 | 215 | 54.70 | 3.54 |  | 0.061 | 0.56 | 1.15 (-0.05:2.36) |
| Nasal bridge length | 98 | 49.62 | 3.90 | 215 | 47.06 | 3.47 | Age* | **0.001** | 0.71 | 1.97 (0.76:3.18) |
| Labial fissure width | 98 | 50.01 | 3.74 | 215 | 47.09 | 3.23 | Height*** | **0.015** | 0.88 | 1.39 (0.27:2.51) |
| Philtrum width | 98 | 13.52 | 1.70 | 215 | 11.78 | 1.65 |  | **<0.001** | 1.05 | 1.45 (0.89:2.00) |
| Philtrum length | 98 | 14.94 | 2.73 | 215 | 13.51 | 2.48 | Height* | 0.124 | 0.56 | 0.67 (-0.18:1.53) |
| Upper lip height | 98 | 21.83 | 2.89 | 215 | 20.07 | 2.74 | Height** | 0.098 | 0.64 | 0.78 (-0.15:1.71) |
| Lower lip height | 97 | 18.87 | 2.45 | 214 | 17.59 | 2.37 |  | **0.018** | 0.53 | 0.98 (0.17:1.79) |
| Upper vermilion height | 98 | 8.43 | 1.75 | 215 | 7.79 | 1.38 | Height* | 0.253 | 0.43 | 0.29 (-0.21:0.80) |
| Lower vermilion height | 98 | 9.56 | 2.18 | 214 | 9.46 | 1.86 |  | 0.417 | 0.06 | -0.27 (-0.94:0.39) |
| Cutaneous lower lip height | 97 | 11.33 | 2.61 | 214 | 9.92 | 2.25 |  | **<0.001** | 0.60 | 1.50 (0.70:2.30) |

^a^ only left variable included. ^b^ Significance of covariates indicated by * ≤ 0.05, ** ≤ 0.01, *** ≤ 0.001. d = Cohen’s d effect size measure (see text for details). Mean diff = signed difference in mm between covariate-adjusted male and female means. All mean and SD values in mm.

**Table S6**. Descriptive statistics and detailed ANCOVA results for the adult age group.

|  | Males | | | Females | | | Statistics | | | |
| --- | --- | --- | --- | --- | --- | --- | --- | --- | --- | --- |
| Variable | N | Mean | SD | N | Mean | SD | Covariates ^b^ | p | d | Mean diff (95%CI) |
| Maximum cranial width | 211 | 152.01 | 5.39 | 349 | 145.72 | 5.11 |  | **<0.001** | 1.20 | 5.55 (4.19:6.90) |
| Minimum frontal width | 213 | 112.54 | 8.54 | 349 | 105.91 | 8.49 | Height***, Age*** | **0.002** | 0.80 | 3.48 (1.33:5.63) |
| Maximum facial width | 213 | 136.66 | 6.14 | 330 | 129.15 | 5.31 | Height*** | **<0.001** | 1.34 | 5.68 (4.22:7.14) |
| Mandibular width | 209 | 103.88 | 5.54 | 343 | 96.55 | 5.88 | Height*** | **<0.001** | 1.21 | 4.95 (3.37:6.53) |
| Maximum cranial length | 212 | 198.05 | 6.19 | 349 | 187.00 | 6.22 | Height*** | **<0.001** | 1.84 | 7.30 (5.74:8.85) |
| Cranial base width | 219 | 147.14 | 5.32 | 353 | 138.48 | 4.97 | Height***, Age* | **<0.001** | 1.74 | 6.23 (4.95:7.51) |
| Upper facial depth ^a^ | 220 | 125.87 | 5.15 | 354 | 118.22 | 4.73 | Height*** | **<0.001** | 1.62 | 4.62 (3.41:5.84) |
| Middle facial depth ^a^ | 220 | 131.68 | 5.21 | 354 | 123.16 | 4.88 | Height*** | **<0.001** | 1.76 | 5.44 (4.20:6.69) |
| Lower facial depth ^a^ | 216 | 150.80 | 6.13 | 346 | 138.66 | 6.05 | Height*** | **<0.001** | 2.10 | 7.79 (6.29:9.29) |
| Morphological facial height | 217 | 126.58 | 6.44 | 352 | 118.35 | 6.11 | Height*** | **<0.001** | 1.58 | 4.08 (2.54:5.62) |
| Upper facial height | 222 | 77.68 | 4.46 | 359 | 74.11 | 4.53 | Height*** | **0.043** | 0.81 | 1.15 (0.04:2.27) |
| Lower facial height | 217 | 72.29 | 5.26 | 352 | 66.45 | 4.97 | Height*** | **<0.001** | 1.17 | 3.36 (2.09:4.65) |
| Intercanthal width | 221 | 33.01 | 3.01 | 359 | 31.25 | 2.83 |  | **<0.001** | 0.61 | 1.38 (0.64:2.12) |
| Outercanthal width | 216 | 88.92 | 3.88 | 357 | 85.42 | 3.75 | Height*** | **0.001** | 0.94 | 1.68 (0.72:2.63) |
| Palpebral fissure length ^a^ | 216 | 28.56 | 1.80 | 357 | 27.70 | 1.95 | Height*** | 0.442 | 0.46 | 0.19 (-0.29:0.67) |
| Nasal width | 220 | 35.99 | 2.48 | 360 | 32.35 | 2.21 |  | **<0.001** | 1.57 | 3.27 (2.68:3.86) |
| Subnasal width | 221 | 19.33 | 2.64 | 360 | 17.14 | 2.21 |  | **<0.001** | 0.93 | 1.95 (1.34:2.56) |
| Nasal protrusion | 222 | 21.05 | 1.94 | 360 | 19.88 | 1.90 | Height***, Age* | **0.040** | 0.62 | 0.51 (0.02:0.99) |
| Nasal ala length ^a^ | 220 | 35.15 | 2.02 | 359 | 31.46 | 1.80 | Height*** | **<0.001** | 2.00 | 2.77 (2.30:3.24) |
| Nasal height | 222 | 56.74 | 3.68 | 360 | 54.49 | 3.92 | Height*** | 0.335 | 0.57 | 0.47 (-0.49:1.43) |
| Nasal bridge length | 222 | 49.76 | 3.68 | 360 | 47.30 | 3.60 | Height*** | **0.019** | 0.69 | 1.10 (0.18:2.01) |
| Labial fissure width | 222 | 50.62 | 3.75 | 360 | 48.00 | 3.31 | Height**, Age** | **<0.001** | 0.76 | 1.65 (0.78:2.52) |
| Philtrum width | 221 | 13.09 | 1.70 | 360 | 11.77 | 1.60 |  | **<0.001** | 0.81 | 1.11 (0.69:1.53) |
| Philtrum length | 221 | 15.68 | 2.59 | 360 | 14.01 | 2.42 |  | **<0.001** | 0.68 | 1.29 (0.66:1.92) |
| Upper lip height | 222 | 22.03 | 2.84 | 359 | 20.47 | 2.53 | Height** | **0.033** | 0.59 | 0.73 (0.06:1.40) |
| Lower lip height | 218 | 19.10 | 2.93 | 358 | 17.69 | 2.31 | Height** | **0.022** | 0.55 | 0.76 (0.11:1.41) |
| Upper vermilion height | 221 | 7.64 | 1.84 | 359 | 7.56 | 1.37 | Height*** | **0.022** | 0.05 | -0.46 (-0.86:-0.07) |
| Lower vermilion height | 222 | 8.99 | 2.18 | 358 | 8.88 | 1.87 | Height*** | **0.042** | 0.05 | -0.52 (-1.03:-0.02) |
| Cutaneous lower lip height | 218 | 12.20 | 2.55 | 358 | 10.70 | 2.23 |  | **<0.001** | 0.64 | 1.27 (0.67:1.87) |

^a^ only left variable included. ^b^ Significance of covariates indicated by * ≤ 0.05, ** ≤ 0.01, *** ≤ 0.001. d = Cohen’s d effect size measure (see text for details). Mean diff = signed difference in mm between covariate-adjusted male and female means. All mean and SD values in mm.

**Figures S1 – S29:** Plots of unadjusted means and associated 95% confidence intervals for each linear distance as a function of sex and age.
